# Polarized macrophages regulate fibro/adipogenic progenitor (FAP) adipogenesis through exosomes

**DOI:** 10.1186/s13287-023-03555-6

**Published:** 2023-11-07

**Authors:** Mengyao Liu, Martin Ng, Tuan Phu, Laura Bouchareychas, Brian T. Feeley, Hubert T. Kim, Robert L. Raffai, Xuhui Liu

**Affiliations:** 1 Department of Veterans Affairs, San Francisco Veterans Affairs Medical Center, San Francisco, CA 94158 USA; 2 Department of Orthopedic Surgery, University of California, San Francisco, 1700 Owens Street, San Francisco, CA 94158 USA; 3 Department of Surgery, Division of Endovascular and Vascular Surgery, University of California, San Francisco, 4150 Clement Street, San Francisco, CA 94121 USA; 4 College of Medicine, California Northstate University, Elk Grove, CA 95757 USA

**Keywords:** Macrophage polarization, Exosomes, Fibro/adipogenic progenitors, Beige fat differentiation, Muscle fatty infiltration, Rotator cuff injury

## Abstract

**Background:**

Macrophage polarization has been observed in the process of muscle injuries including rotator cuff (RC) muscle atrophy and fatty infiltration after large tendon tears. In our previous study, we showed that fibrogenesis and white adipogenesis of muscle residential fibro/adipogenic progenitors (FAPs) cause fibrosis and fatty infiltration and that brown/beige adipogenesis of FAPs promotes rotator cuff muscle regeneration. However, how polarized macrophages and their exosomes regulate FAP differentiation remains unknown.

**Methods:**

We cultured FAPs with M0, M1, and M2 macrophages or 2 × 10^9^ exosomes derived from M0, M1 and M2 with and without GW4869, an exosome inhibitor. In vivo, M0, M1, and M2 macrophages were transplanted or purified macrophage exosomes (M0, M1, M2) were injected into supraspinatus muscle (SS) after massive tendon tears in mice (*n* = 6). SS were harvested at six weeks after surgery to evaluate the level of muscle atrophy and fatty infiltration.

**Results:**

Our results showed that M2 rather than M0 or M1 macrophages stimulates brown/beige fat differentiation of FAPs. However, the effect of GW4869, the exosome inhibitor, diminished this effect. M2 exosomes also promoted FAP Beige differentiation in vitro. The transplantation of M2 macrophages reduced supraspinatus muscle atrophy and fatty infiltration. In vivo injections of M2 exosomes significantly reduced muscle atrophy and fatty infiltration in supraspinatus muscle.

**Conclusion:**

Results from our study demonstrated that polarized macrophages directly regulated FAP differentiation through their exosomes and M2 macrophage-derived exosomes may serve as a novel treatment option for RC muscle atrophy and fatty infiltration.

**Supplementary Information:**

The online version contains supplementary material available at 10.1186/s13287-023-03555-6.

## Introduction

Rotator cuff (RC) tears are the most common upper extremity cause for musculoskeletal physician visits. Up to 20% of patients greater than the age of 50 have evidence of symptomatic rotator cuff tears and 49% of patients have been found to have cuff tears over the age of 70 [1]. Though patients with small RC tear repairs usually have significant improvement of shoulder function after surgery [2], large or massive RC repairs are accompanied with high re-tearing rates and sub-optimal clinical outcomes. Failures of attempted large RC repairs are at least partially related to the degeneration of RC muscles, a process mediated through muscle atrophy, fatty infiltration, and fibrosis [3–5]. Clinical studies have demonstrated that muscle atrophy and fatty infiltration (FI) are independent factors responsible for poor clinical outcomes after surgical repair [6–8]. However, there is no effective treatment for RC muscle atrophy and fatty infiltration available at this time due to our limited understanding of the underlying mechanisms regarding muscle pathophysiology following tendon tears.

Evidence from chronic muscle pathologies suggests that inflammation, particularly mononuclear phagocyte (MP) infiltration, contributes to muscle degeneration [9]. Pro-inflammatory cytokines such as TNFα and IL-6 stimulate apoptosis and catabolism of myocytes, thus causing muscle atrophy as seen in cancer cachexia and autoimmune disorders [9, 10]. Previous animal studies have shown increased macrophage recruitment with muscle atrophy and fatty infiltration after tendon tears [11, 12]. Recent clinical studies confirmed that the infiltration of macrophages was accompanied by increased inflammatory cytokine levels in RC muscles with full thickness tendon tears [13, 14]. Moreover, macrophages adopt different functional programs through the process of polarization in response to the different environmental signals. According to their polarization status, the phenotypes of the macrophage have been divided into 2 groups: M1 (classically activated macrophages) and M2 (alternatively activated macrophages) [15]. A previous study showed that chronic muscle degeneration in Duchenne muscular dystrophy is partially caused by the classical pro-inflammatory M1 macrophages [16]. A recent quantitative analysis of immune cells in RC muscles showed a significantly increased number of M1 and M2 macrophages that accompany muscle atrophy and fatty infiltration after tendon injury [17]. Our previous study showed that muscle fibro/adipogenic progenitors (FAPs) are the cellular source for RC muscle fibrosis and fatty infiltration [18]. However, the relationship between macrophage infiltration and FAP fibro/adipogenesis remains unknown.

The discovery of intercellular signaling properties of extracellular vesicles (EVs) has opened up new avenues in investigating intercellular communication. Exosomes are a type of EV that originate within multivesicular endosomal compartments (MVB) and display a size between 30 to 100 nm [19]. Exosomes are generated by many types of cells including adaptive immune cells and macrophages [20]. They contain numerous cargo molecules, including mRNA, micro-RNA, long non-coding RNAs (lncRNAs), proteins, and lipids [21–24]. Recent work from our laboratory showed that macrophage-derived exosomes regulate hematopoiesis and inflammation in atherosclerosis [25]. Exosomes have been shown to be the mediator in regulating adipogenic differentiation in mesenchymal stem cells [26]. However, the role of macrophage-derived exosomes in the regulation of FAP adipogenic differentiation remains unknown. The goal of this study was to define the role of polarized macrophages (M1 and M2) and their exosomes in the regulation of FAP differentiation, RC muscle atrophy, and fatty infiltration in a mouse model. We hypothesized that M1 macrophages promote the process of FAP adipogenesis, RC muscle atrophy, and fatty infiltration, while M2 macrophages inhibit the process of FAP adipogenesis, RC muscle atrophy, and fatty infiltration. We further hypothesized that this effect is mediated through exosomes produced by the different forms of macrophages.

## Materials and methods

### Muscle digestion and FAP isolation

FAPs were isolated from SS muscles in C57BL/6 J wildtype mice at 3 months (*N* = 4). In order to evaluate FAP BAT differentiation, additional FAPs were isolated from SS muscles in healthy UCP1 reporter mice (Ucp1-luc2-tdTomato)1Kajim/J (*N* = 4). In brief, SS muscles were minced into 1 mm small pieces with sterile scissors in a cell culture hood. The mixture was incubated with 0.2% Collagenase II for 90 min in 37 °C sterile water bath. Washing buffer (F/10, 10% Horse Serum, 1 × HEPES) was added into the mixture and centrifuged at 1500 rpm for 5 min at room temperature. The supernatant was then transferred to a new 50 mL centrifuge tube. The remnant was rinsed with washing buffer and spun down at 1500 rpm for 5 min. The newly collected supernatant was combined with the supernatant from the last round. D2 solution (0.06% Collagenase II, 0.15% Dispase with washing buffer) was added and incubated for 30 min at 37 °C. The solutions were then passed through a 70-μm cell strainer (VWR International) followed by a 40-μm cell strainer (VWR International). The filtered cells were washed with 40 mL of FACS buffer (2.5%FBS, 20 mM EDTA in PBS) and spun down at 1500 rpm for five minutes. The supernatant was then discarded, and the cell pellets were resuspended with 500μL of FACS buffer. The cells were incubated with anti-CD31-FITC (BD bioscience, Clone 390), anti-CD45-FITIC (BD bioscience, Clone WM59), anti-integrin α7-APC (R&D systems, Clone #334908) and PE-Cy7-Sca1 (BD bioscience, Clone. E13-161.7) and anti-CD140a-BV421 (BD bioscience, Clone APA5) for 60 min before being sorted with FACSAria™ II (BD bioscience). The FAPs were collected as the CD31-/CD45-/ITGA7-/Sca1 + /CD140a + cell population (Additional file 1: Fig. S1) [27].

### FAP culture and characterization

After sorting, the FAPs were seeded into 1% Matrigel pre-coated 24-well plates (1 h at room temperature) at a density of 5,000 cells per well. The FAPs cells were cultured for one week with standard cell culture medium (SM) (Ham’s F-10, 10% fetal bovine serum, 10 ng/ml bFGF and 1% antibiotic–antimycotic solution, Thermo Fisher Scientific, MA USA). FAPs attached to the plates after one to three days. After seven days, FAPs proliferated to 50–70% confluency within the 24-well plate. With adipogenic differentiation media, FAPs differentiated into white adipose cells (perilipin A positive, UCP-1 negative) and brown adipose tissue (Perilipin A positive, UCP-1 positive) in 14 days. With fibrogenic differentiation media, FAPs differentiated into fibroblast (Col1A and aSMA positive, UCP-1 negative, perilipin A negative). For adipogenic differentiation, the cells were cultured with adipogenic differentiation medium (100 μM IBMX, 2.5 μM DEX, 100 mM Indomethacin, 10 μg/ml insulin) for 2 weeks. For the exosome treatment, 2 × 10^9^ exosomes derived from macrophages (M0, M1, M2) were added into the FAP cell culture medium. Fresh exosomes were added every other day for a course of 14 days.

### Macrophage culture and polarization

Murine macrophages (Raw 264.7, ATCC #TIB-71) were cultured with DMEM/F-12 with 10% FBS and 1% penicillin–streptomycin (GIBCO, CA, USA). To polarize the macrophages into the M1 subtype, the macrophages were treated with 100 ng/ml LPS with 50 ng/ml IFNγ for 7 days; to polarize the macrophages into the M2 subtype, macrophages were treated with 10 ng/ml IL-4 and IL-10 in DMEM/F-12 for 7 days. M1 macrophages were quantified by flow cytometry as cd11b + cd68 + cd11c + /cd206 − cells and M2 macrophages were quantified by flow cytometry as cd11b + cd68 + cd11c−/cd206 + cells. Unpolarized macrophage M0 were defined as cd11b + cd68 + cells as previously described [28].

### Macrophage exosome isolation

Exosomes secreted by M0, M1, and M2 bone marrow-derived macrophages were purified using the recently described cushioned-density gradient ultracentrifugation (C-DGUC) method [29]. The bone marrow-derived macrophages originated from the bone marrow of C57BL/6 J (*n* = 3) mice and were induced by M-CSF for 7 days. Macrophages were then differentiated into M1 and M2 cells, the medium was collected, and the exosomes were isolated [25]. Briefly, cell culture supernatant was collected from the macrophages with an average viability of 95%. To avoid exosome contamination from FBS, exosome depleted FBS (Thermo Fisher, CA, USA) was used for macrophage culturing. The supernatant was centrifuged at 400 g for 10 min at 4 °C to pellet dead cells and debris, followed by centrifugation at 2000 *g* for 20 min at 4 °C to eliminate debris and large vesicles. The supernatant was then filtered with 0.2 μm filter paper and centrifuged on a 60% iodixanol cushion (Sigma-Aldrich, MO, USA) at 100,000 *g* for 3 h (Type 45 Ti, Beckman Coulter, IN, USA). OptiPrep density gradient (5%, 10%, 20% w/v iodixanol) was employed to further purify exosomes at 100,000 *g* for 18 h at 4 °C (SW 40 Ti rotor). Afterward, twelve 1 mL fractions were collected starting from the top of the tube. Fraction seven of the gradient was dialyzed in PBS with the Slide-A-Lyzer MINI Dialysis Device (Thermo Fisher Scientific, CA, USA) and used for subsequent experiments and analyses. Exosome size and concentration measured with NanoSight LM14 (Malvern Instruments, Westborough, MA, USA) and a 488-nm detection wavelength was used. The detailed characterization is described in the Additional file 2: Fig. S2. All the exosomes were stored at 4 °C and used within one month after isolation.

### Macrophage coculture treatment

The FAPs were seeded in 24-well plates with a density of 0.1 million per well (*n* = 6 for each macrophage condition), and 250,000 macrophages were seeded into the transwell culture inserts (cat# 3413, corning, IL, USA). Both cells were cocultured with the same F-10 medium with 10% FBS and 1% antibiotic–antimycotic for 14 days. FAPs were fixed with PFA and stained by perilipin A and UCP1. The macrophages were then fixed with PFA and stained with cd11b and cd202. The polarization efficiency was quantified using flow cytometry. For the exosome inhibition experiment, 1 μM GW4869 (cat # D1692, Sigma, CA, USA) was used and 0.1% DMSO was used as the control group.

### Rotator cuff transection procedure

PDGFRα-GFP FAP reporter mice (3 months old, *N* = 24, Jackson laboratory Corp., Sacramento, CA, Stock # 007669) underwent unilateral rotator cuff tear surgery with complete supraspinatus and infraspinatus tendon transection plus suprascapular denervation transection (TT + DN) as described previously [30]. A sham surgery, in which the tendons and nerves were exposed but not transected, was performed on the contralateral side. This sham surgery procedure has been proven to not cause rotator cuff muscle atrophy or fatty infiltration [31]. The PDGFRα-GFP FAP mice were sacrificed at 1, 2, and 6 weeks after surgery and supraspinatus muscles from both RC tear and sham side were harvested for histology (*n* = 5 in each group). Intact rotator cuff tendons and suprascapular nerves were verified at the side with sham surgery. All surgeries were done under anesthesia using an inhalant anesthetic. For the inhalant anesthetic, mice were first placed in the chamber with 1L oxygen/minute with 5% isoflurane until mice were immobile and breathing deep and slow. Mice were then taken out from the chamber and put on the bench with a nose mask and administered 1L oxygen /minute and 2% isoflurane during surgery.

### Macrophage transplantation

Adult female wildtype C57BL/6 J mice (*n* = 16, Jackson laboratory Corp., Sacramento, CA, Stock # 000664) and adult female PDGFRα-GFP reporter mice (*n* = 16, Jackson laboratory Corp., Sacramento, CA, Stock # 0007669) underwent unilateral rotator cuff tear surgery at 3 months old with complete supraspinatus and infraspinatus tendon transection plus suprascapular denervation transection (TT + DN) as described previously [30]. Polarized M1 and M2 macrophages with 10μL of Phosphate Buffer solution (PBS) and 250,000 uninduced M0 cells were mixed with 10μL of Matrigel and transplanted into the supraspinatus muscle on the same day of Rotator cuff injury. The control group included 10μL of PBS with 10μL Matrigel at the time of surgery. The mice were sacrificed at six weeks after surgery when fatty infiltration became obvious as described in the previous study [30]. In each group, C57BL/6 J mice (*n* = 4) were used for histologic analysis and PDGFRα-GFP reporter mice (*n* = 4) were used for immunochemistry. The mice were sacrificed at six weeks after injury because our previous studies have determined that it was a key time point in determining FAP differentiation [18]. The SFVAMC Institutional Animal Care and Use Committee (IACUC) approved all procedures and handling of the animals. The detailed schematic flow of the experiment design is included in the Additional file 3: Fig. S3.

### Macrophage exosome injection

Adult female wildtype C57BL/6 J mice (*n* = 16, Jackson laboratory Corp., Sacramento, CA, Stock # 000664) underwent unilateral massive rotator cuff tear surgery at 3 months old with complete supraspinatus and infraspinatus tendon transection plus suprascapular denervation transection (TT + DN) as described previously [31]. The mice were then randomly divided into four groups (four mice in each group). Injection into supraspinatus muscle was performed with 2 × 10^9^ of M0, M1, and M2 exosomes dissolved in PBS. The control group used 20μL of PBS at the time of surgery. Mice were sacrificed at 6 weeks after surgery when fatty infiltration became obvious based on previous experience [18]. S1.

### Muscle harvesting and histology

Bilateral supraspinatus (SS) muscles were harvested, and the remaining tendon and scar tissue were removed at the muscle/tendon junction after mice were sacrificed. The muscles were then snap frozen in liquid nitrogen-cooled isopentane and sectioned at a thickness of 10 μm with a cryostat. Oil-red-O (Sigma-Aldrich, Burlington, MA) staining was conducted in order to evaluate the FI in muscle section as described previously [14]. Trichrome staining was conducted to evaluate the fibrosis index. After fixing in 4% paraformaldehyde and rinsing in PBS, sections were incubated in blocking solution (0.1% Triton X-100, 5% bovine serum albumin in PBS) for 1 h and then incubated with the primary antibodies for immunostaining (anti-Laminin, L9393, Sigma-Aldrich, 1:500 diluted; anti-UCP1, sc-6529, Santa Cruz Biotechnology, 1:50 diluted) at 4° C overnight. After PBS rinsing, the sections were treated with secondary antibodies (1: 5000 diluted) for 2 h. For PDGFRα-GFP reporter mice, secondary antibodies of Alexa Fluor 594-conjugated anti-rabbit IgG (ab150076) and Alexa Fluor 647-conjugated anti-goat IgG (ab150131) were used. For C57BL/6 J mice, secondary antibodies of Alexa Fluor 488-conjugated anti-rabbit IgG (ab150077) and Alexa Fluor 594-conjugated anti-goat IgG (ab150140) were used. After a PBS rinse, slides were mounted with VectaShield plus DAPI and a coverslip. Histologic pictures were taken with the Zeiss digital camera on an Axio Imager 2 microscope (Zeiss). The fat area fraction was evaluated by dividing the Oil-Red-O-stained area by the entire sample [14]. The collagen area fraction was evaluated by dividing the collagen-stained area by the entire sample [14].

### Real-time qPCR

The total cell RNA was isolated using Trizol reagent (Invitrogen, Inc., Carlsbad, CA, USA) according to manufacturer’s instructions. One microgram of total RNA was used to generate cDNA using Maxima First Strand cDNA Synthesis Kit (Roche Applied Bioscience, Indianapolis, IN, USA) for each sample. Real-time qPCR was run with Fast SYBR Green Master Mix (Applied Biosystems, CA, USA) on a Viia7 Real-Time Detection System (Applied Biosystems, CA, USA). Expression levels of interested genes were normalized with the housekeeping gene of ribosomal protein S26 and compared across samples with ΔΔCt method [32]. Six biological replicates were included in each group. The amount of RNA used in this experiment was 1000 ng. The primer sequences used in this experiment are listed in Additional file 4: Table S1. All data was presented in the form of mean ± standard deviation. Statistical analyses were performed using the ANOVA test with significance at *p* < 0.05 [33]. Tukey’s honest significant difference (HSD) post hoc test was used for this experiment.

**Fig. 1:**
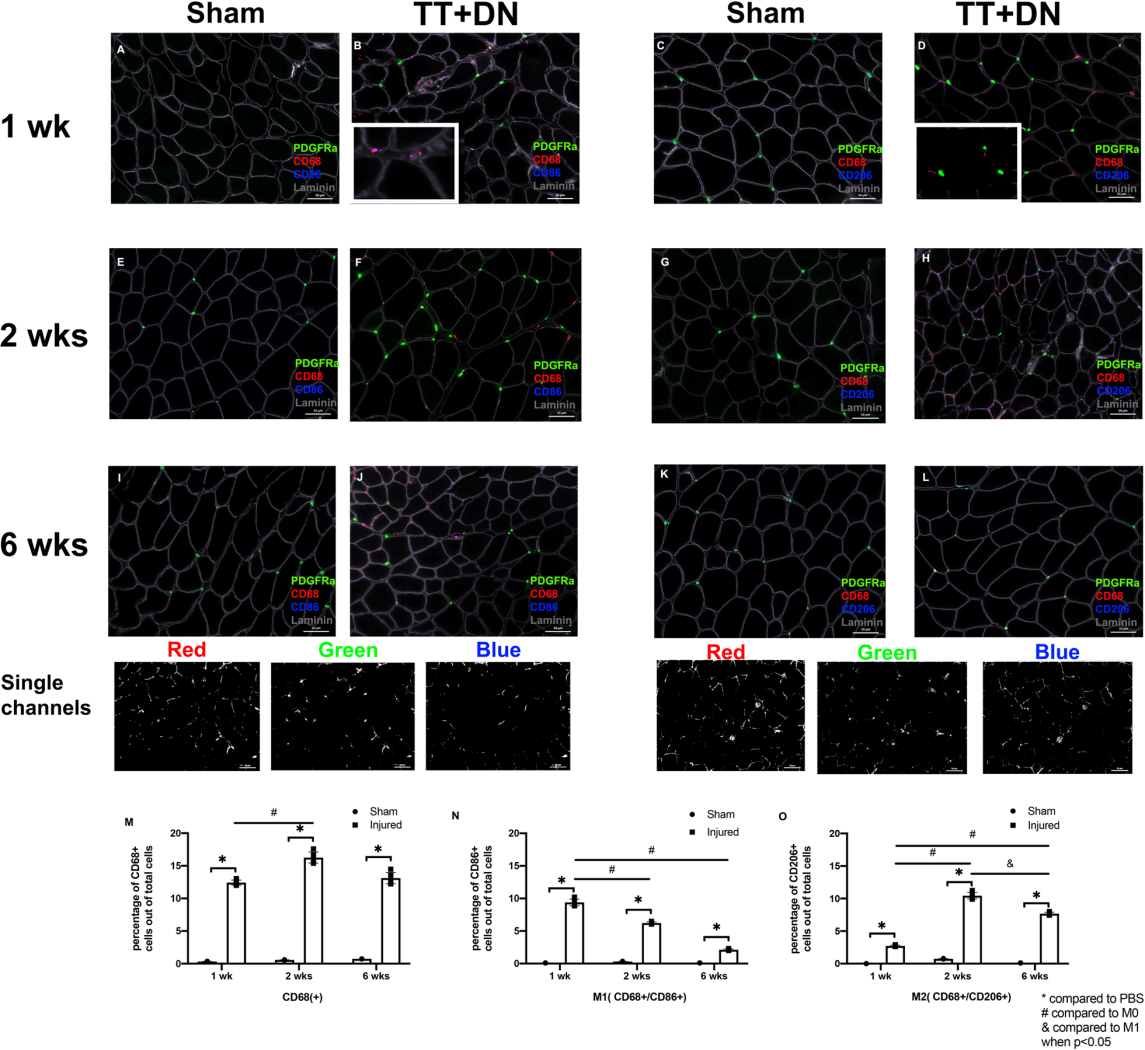
**A**–**L** The macrophage proliferation profile after TT + DN injury. Immunofluorescent staining indicated that when compared to the sham group, the number of macrophages were significantly increased in the TT + DN groups after 1 week, 2 weeks, and 6 weeks of injury. **M** The percentage of macrophages (cd68 + cell number/ total cells × 100%) significantly increased at 1 week, 2 weeks, and 6 weeks after TT + DN (vs. sham) (^ compared to sham side * compared to 1 week, # compared to 2 weeks when *p* < 0.05). **N** The percentage of M1 was quantified using the formula (cd86 + &cd68 +)/ total cd68 +  × 100% (* indicates *p* < 0.05). O) The percentage of M2 was quantified using the formula (cd206 + &cd68 +)/ total cd68 +  × 100% (^ compared to sham side * compared to 1 week, # compared to 2 weeks when *p* < 0.05)

### Immunostaining

In order to evaluate FAP adipogenesis, FAPs were stained using Goat anti-mouse perilipin A (Cat#ab61682, 1:1000, Abcam, CA, USA) as a mature adipocyte marker, pre-conjugated mouse anti-mouse αSMA-Cy3 (alpha smooth muscle actin Cy3, clone 1A4, 1:500, sigma-Aldrich, MO, USA) as a fibrosis marker and rabbit anti-mouse UCP1 (Cat#ab10983, brown fat marker, 1:1000, Abcam, CA, USA) as a brown adipose tissue marker at all time points overnight. The FAPs were then stained with Donkey anti-Goat FITC (Cat#ab150129, 1:2000, Abcam, CA, USA), Donkey anti Rabbit Cy5 (Cat#ab150075, 1:2,000, Abcam, CA, USA), and DAPI (1:10,000, Sigma-Aldrich, MO, USA). Three replicates per condition were included. The adipogenesis index was determined by dividing the number of cells expressing perilipin by the total number of nuclei in each image.

### Image capture and quantification

To measure the contribution of macrophages after TT + DN injury, whole-section images for each muscle were captured at 360 nm (DAPI), 480 nm (GFP), and 570 nm (rhodamine) using an Axio Observer D1 fluorescence microscope. Using image analysis software, color channels were split into blue, green, and red. DAPI^+^, GFP^+^ (cd86-M1 or cd206-M2) and rhodamine (cd68)^+^ cells were quantified in each muscle sample. To determine GFP/rhodamine double positive cells, images for green and red channels in each sample were overlapped. GFP^+^ (cd86-M1 or cd206-M2), rhodamine^+^ and GFP^+^/rhodamine^+^ cells were quantified manually in each section by two blinded reviewers. The percentage of GFP and rhodamine double positive cells within all rhodamine^+^ cells in each muscle was calculated as GFP^+^ (cd86-M1 or cd206-M2) and rhodamine (cd68)^+^ cell number/total rhodamine (cd68)^+^ cell number × 100%. For other staining, the positive percentage of cells refers to all the nuclei that overlapped between the staining and DAPI divided by the total amount of DAPI positive in the image. Data were run through two-way ANOVA to compare the percentages of M1 and M2 macrophages and was presented as mean ± standard deviation.

### Statistical analysis

The number of animals required was determined based upon power analysis using our best approximation of true effect and anticipated sample variability using the assumption of *α* = 0.05, *ß* = 0.80, *γ* = 0.8. With these calculations, we concluded that a minimum number of four animals was required in each group. All data were presented in the form of mean ± standard deviation. Statistical analyses were performed using ANOVA test with significance at *p* < 0.05 [33]. Tukey’s honest significant difference (HSD) post hoc test was used for this experiment.

## Results

### *Macrophage proliferation increased in RC muscle after TT* + *DN injury*

Histology analysis showed the percentage of macrophages significantly increased in SS muscle after rotator cuff surgery TT + DN at one week, two weeks, and six weeks post-injury compared to the sham with a peak of M1 at one week and M2 at two weeks (Fig. 1). The percentage of M0 increased significantly at two weeks compared to one week, however the percentage did not decrease significantly at six weeks. The same trend was observed with the percentage of M2 after rotator cuff injury. The percentage of M1 increased at one week significantly; however, the percentage dropped significantly after two weeks and six weeks compared to one week. The ratio of M1 to M2 was 3.65 ± 1.62 at one week, 0.41 ± 0.49 at two weeks, and 0.28 ± 0.22 at six weeks (Fig. 1).

### Macrophage-enhanced FAP adipogenesis

**Fig. 2:**
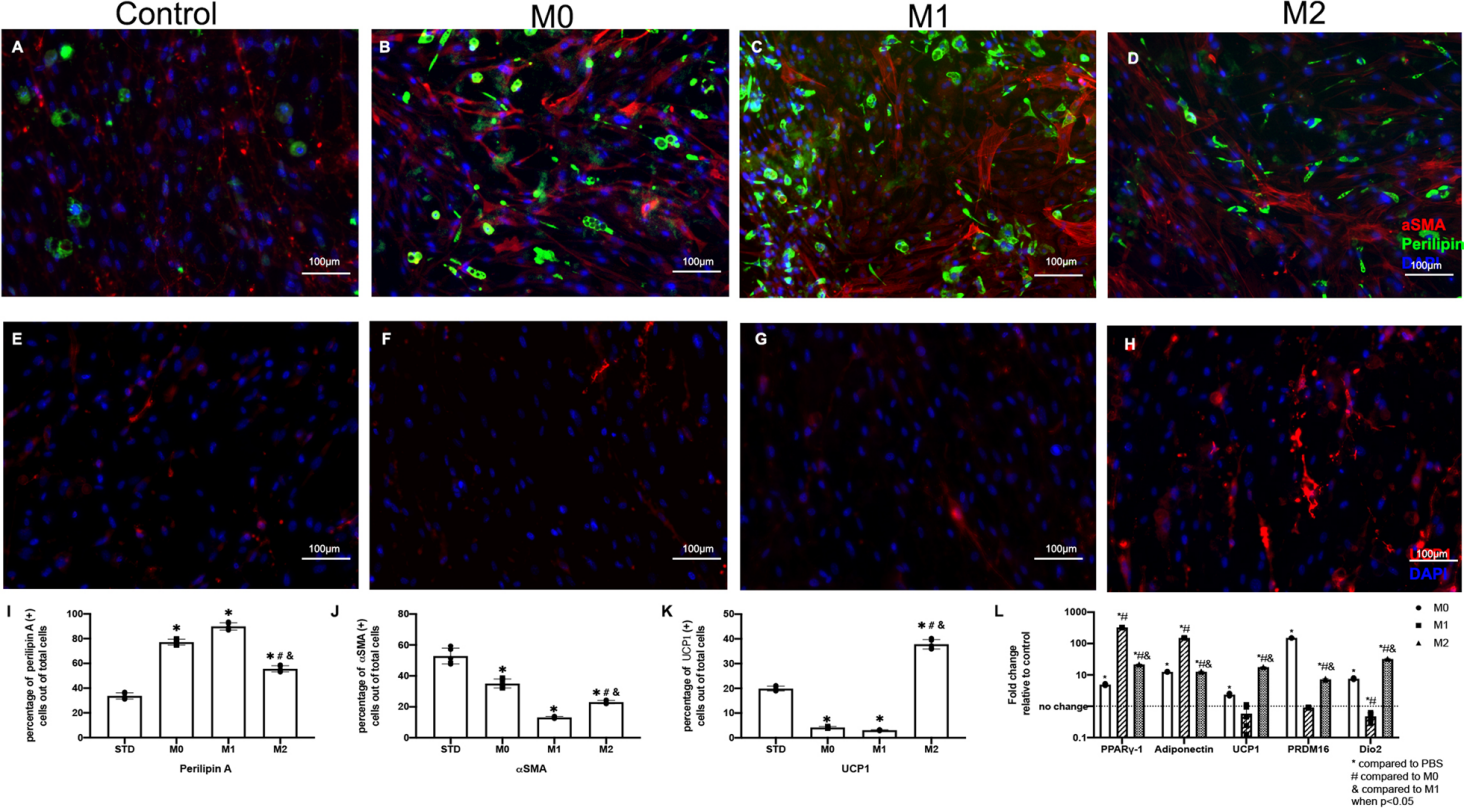
**A**–**D** The perilipin A and αSMA stained FAPs cocultured with macrophages (M0, M1 and M2) (Perilipin A-Green, αSMA-Red, DAPI-Blue). **E**–**H** Typical UCP1 staining of FAPs cocultured with macrophages (M0, M1 and M2) (UCP1-Red, DAPI-Blue). **I** The quantification of perilipin A positive cells out of total cells. **J** The quantification of αSMA positive cells out of total cells. **K** The quantification of UCP1 positive cells out of total cells. **L** The adipogenic marker gene expression of FAPs in each coculture group compared to the control group (* compared to the PBS group; # compared to M0; & compared to M1 when *p* < 0.05)

After FAPs were cocultured with M0, M1, and M2 for 14 days, the percentage of perilipin A ( +) adipocytes increased significantly when compared to the control (Fig. 2A–H). However, there was no significant difference between the M0, M1, and M2 groups. The percentage of UCP1 positive brown/beige fat (BAT) FAPs was significantly increased in the M2 group compared to the M0, M1, and control group (Fig. 2K). Coculture with M1 macrophages significantly decreased UCP1 positive BAT FAPs compared to the STD group (*p* < 0.00001). RT-PCR results showed that the gene expression level of adipogenic markers PPARγ-1 and Adiponectin were significantly increased in the M0, M1, and M2 group compared to the control group. The beige adipogenesis marker panel, also known as UCP1, PRDM16, and DIO2, was significantly increased in the M0 and M2 coculture group, but not changed in the M1 group compared to the control group. These data suggest that the M2 macrophages induce FAP beige adipose differentiation compared to the M0, M1, and control groups.

### GW4869 exosome inhibitor treatment diminished the effects of macrophages

**Fig. 3:**
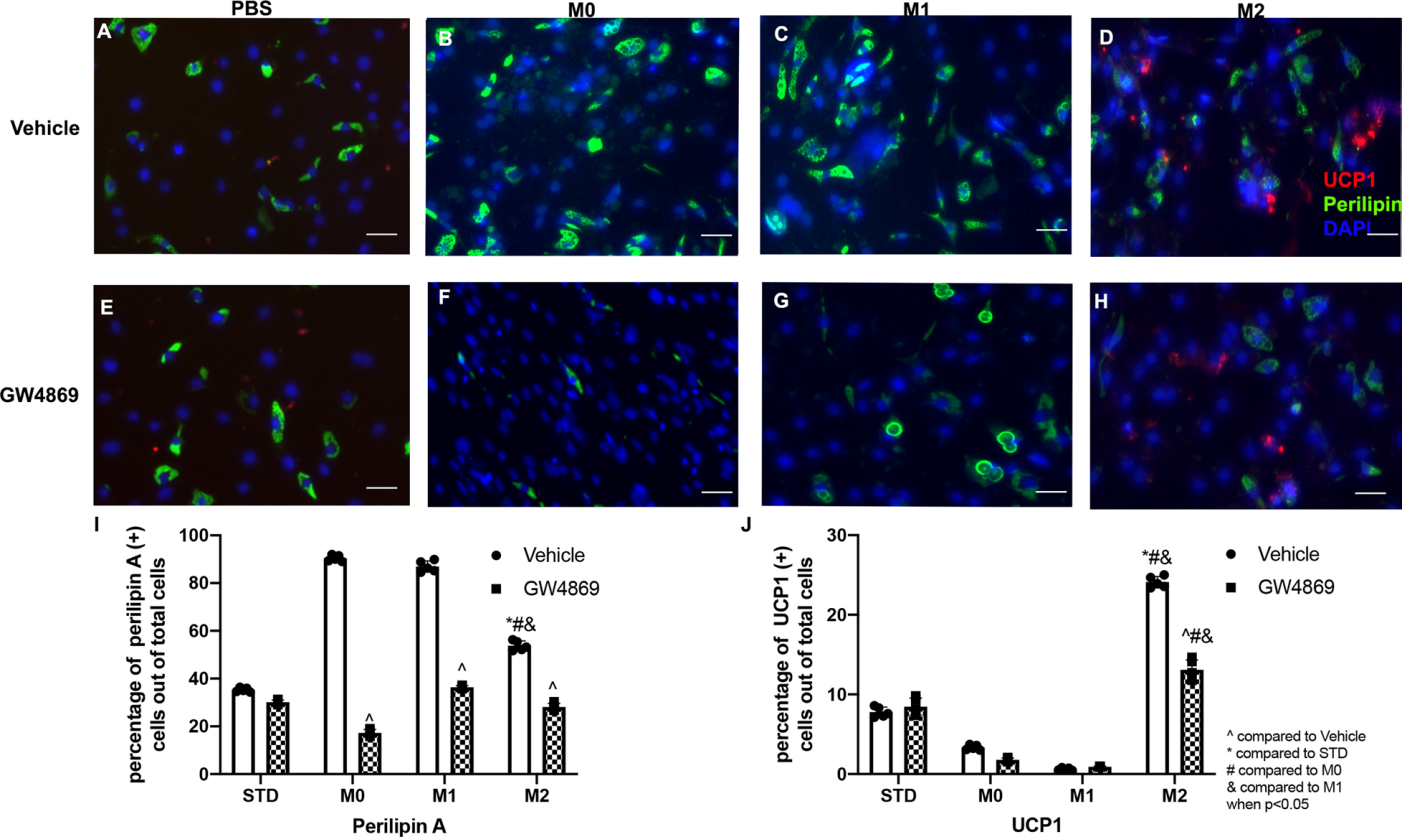
**A**–**D** Typical perilipin A and UCP1 staining of FAPs cocultured with macrophage (UCP1-Red, Perilipin A-Green, DAPI-Blue). **E**–**H** Typical perilipin A and UCP1 staining of FAPs cocultured with macrophage treated with GW4869 (exosome depletion agent) (UCP1-Red, Perilipin A-Green, DAPI-Blue). **I** The quantification of perilipin A positive cells out of the total number of cells. **J** The quantification of UCP-1 positive cells out of the total number of cells (* compared to standard group without macrophage coculturing; # compared to M0; & compared to M1; ^ compared to the vehicle group when *p* < 0.05)

Administration of GW4869 to the cell culture medium substantially diminished the effect of M2 macrophages in inducing FAP adipogenesis (Fig. 3A–H). Compared to the coculture group, the percentage of adipocytes was not significantly changed in the M0, M1, and M2 group when compared to the PBS group. No significant difference was found between the M0, M1, and M2 groups (Fig. 3I). GW4869, an exosome inhibitor (Fig. 3E–H), also diminished the effect of macrophage on FAP BAT differentiation. There was no difference in the percentage of UCP1 positive BAT-FAPs out of total FAPs number found between the M0, M1, M2 and control groups (Fig. 3J). Inhibition of exosomes abolished the role of macrophages in regulating FAP differentiation.

### Macrophage exosome treatment-enhanced FAP adipogenesis

**Fig. 4:**
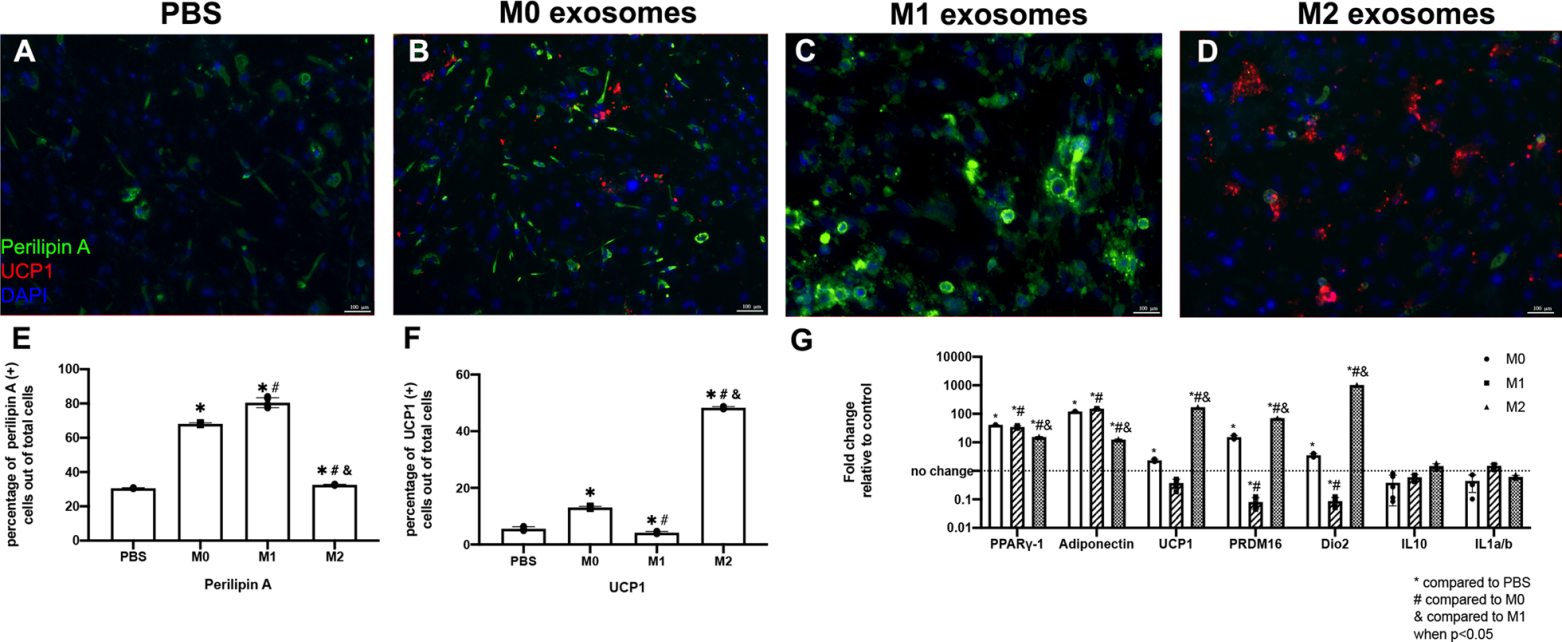
**A**–**D** Typical perilipin A and UCP1 staining of FAPs cocultured with macrophage exosomes (UCP1-Red, Perilipin A-Green, DAPI-Blue). **E** The quantification of perilipin A positive cells out of the total number of cells. **F** The quantification of UCP1 positive cells out of the total number of cells **G** Adipogenic marker gene expression of each coculture group when compared to the control group (*compared to the PBS group, # compared to M0; & compared to M1 when *p* < 0.05)

After FAPs were treated with M0, M1, and M2 exosomes for 14 days, the percentage of perilipin A (+) adipocytes increased significantly in the M0 and M1 group compared to the PBS group. However, no difference in the M2 exosome treatment group was found when compared to the PBS control (Fig. 4). The M1 exosomes significantly improved FAP adipogenesis when compared to the M2 exosomes (*p* < 0.0001) as measured by the percentage of perilipin A (+) FAPs. The M2 macrophage exosomes significantly increased UCP1(+) BAT-FAPs compared to the M0, M1, and PBS groups. M1 macrophage exosomes showed no effect in inducing FAP BAT differentiation as UCP1 expression compared to the control group (*p* < 0.00001). RT-PCR showed that the gene expression level of PPARr-1 and Adiponectin were significantly increased in the M0, M1, and M2 exosome treated groups when compared to the control group. BAT differentiation marker gene expression levels were significantly increased in the M2 and M0 exosome group, but not in the M1 group (except PRDM16) when compared to the PBS group (Fig. 4). M2 macrophage exosomes activated the BAT gene expression and induced FAP beige/brown adipogenesis.

### Macrophage transplantation altered the morphology of muscle after rotator cuff injury

**Fig. 5:**
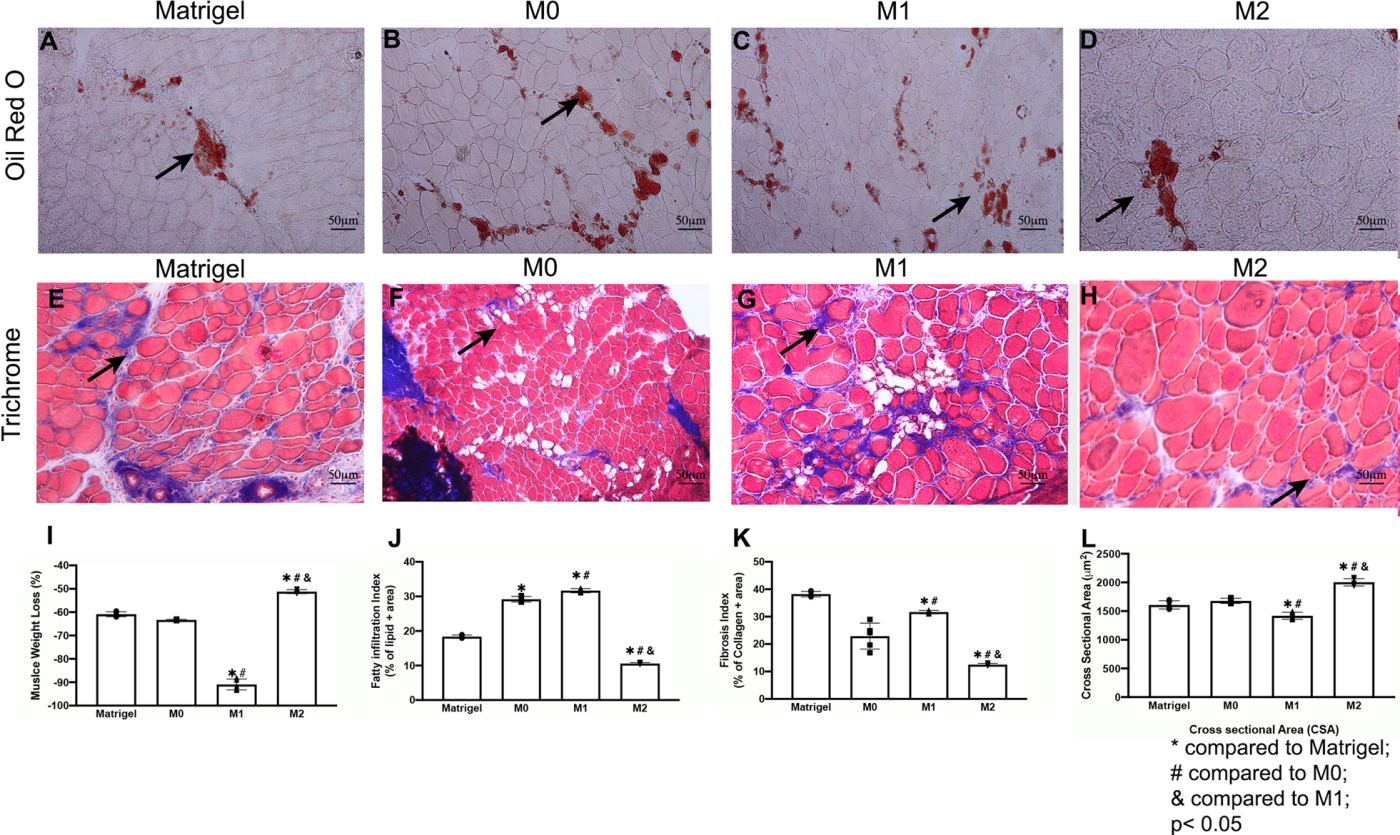
**A**–**D** Macrophage transplantation diminished muscle regeneration after rotator cuff injury (TT + DN) after 6 weeks. It was shown that M2 enhances muscle regeneration after TT + DN surgery. Typical Oil Red O staining images (original magnification × 200) of SS in Matrigel, M0, M1, and M2 transplantation (Lipid droplet—red). **E**–**H** Typical Trichrome staining images of SS in Matrigel, M0, M1, and M2 transplantation (collagen—blue, muscle fiber—red, nuclei—purple). **I** The total muscle weight after rotator cuff injury (TT + DN) at 6 weeks normalized with body weight. Muscle weight loss = ([SS right—SS left]/SS left) × 100%. **J** The quantification of the fatty infiltration index was measured as the percentage of the area of lipids out of the total area of the image. **K** The quantification of the fibrosis index was measured as the percentage of the area of collagen out of the total area of the image. **L** The quantification of myofiber cross-sectional area, mean $\pm$ SD (* compared to PBS; # compared to M0; & compared to M1 when *p* < 0.05)

**Fig. 6:**
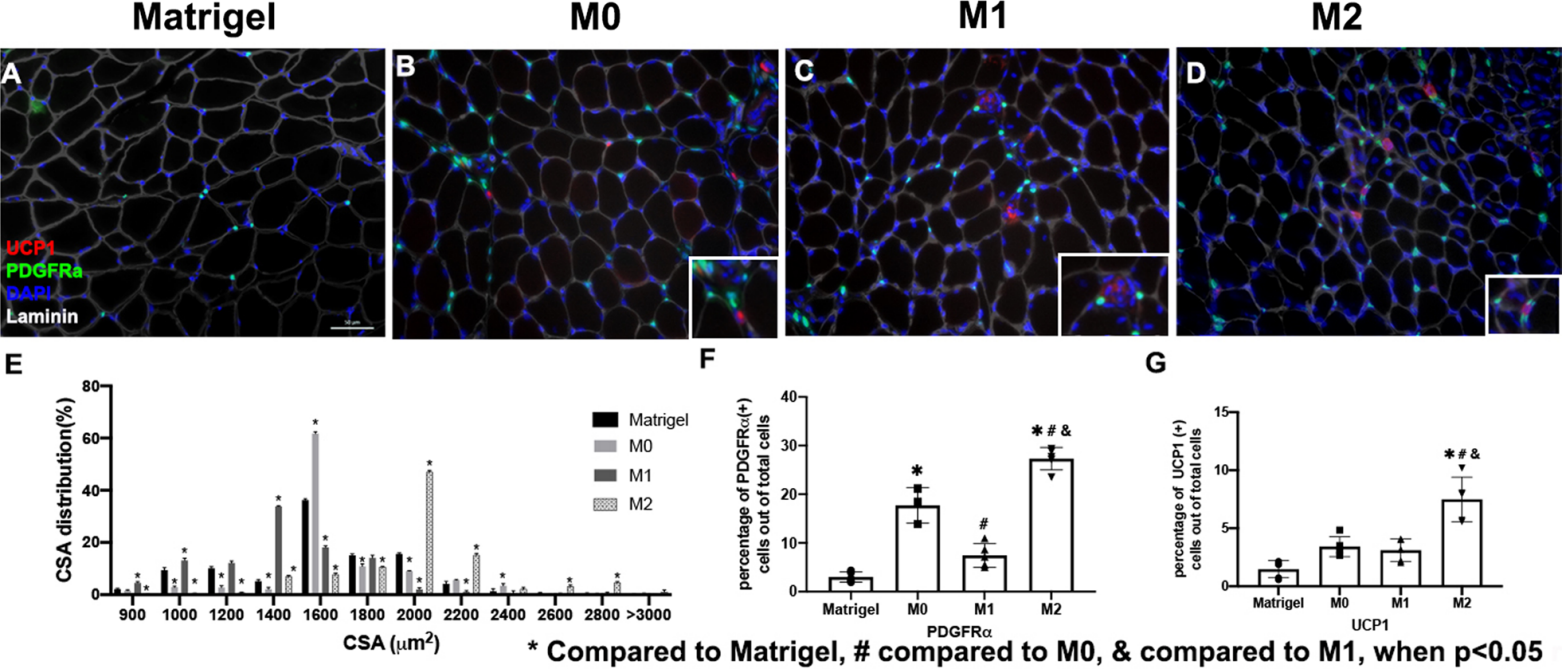
M0 and M1 transplantation diminished muscle regeneration 6 weeks after rotator cuff injury (TT + DN). M2 macrophages transplantation enhanced FAP proliferation after TT + DN injury. **A**–**D** Typical IHC (original magnification × 200) of SS in Matrigel, M0, M1 and M2 transplantation (PDGFRα-Green, UCP1-Red, Laminin-Grey, DAPI-Blue). **E** The muscle fiber size distribution by percentage (CSA um2). **F** The quantification of FAP proliferation was determined by the percentage of PDGFRα-positive cells divided by the total number of cells. **G** The quantification of UCP1 expression with the percentage of UCP1 positive cells out of the total cells (* compared to the Matrigel group # compared to M0 & compared to M1when *p* < 0.05)

Histology analysis showed muscle weight loss, fatty infiltration, and fibrosis significantly decreased in SS muscle after M2 macrophage transplantation in TT + DN at six weeks injury when compared to Matrigel (Fig. 5A–H). Moreover, the cross-sectional area (CSA) significantly improved in SS after M2 exosome transplantation (2002.2 ± 63.9 (M2) vs 1418.7 ± 62.3 (M1) vs 1678.7 ± 45.0 (M0) vs 1607.5 ± 71.8 (Matrigel), Fig. 5L). The overall UCP1 expression was significantly increased in the M2 macrophage transplantation group as well (7.5 ± 1.9 (M2) vs 1.5 ± 0.7 (Matrigel), Fig. 6G). However, the M1 transplantation group showed reduced UCP1 expression (Fig. 5, 6). Transplanting M2 macrophages decreased fatty infiltration, fibrosis, and atrophy and induced UCP1 expression in SS muscle.

### Macrophage exosome injection altered muscle morphology after rotator cuff injury

**Fig. 7:**
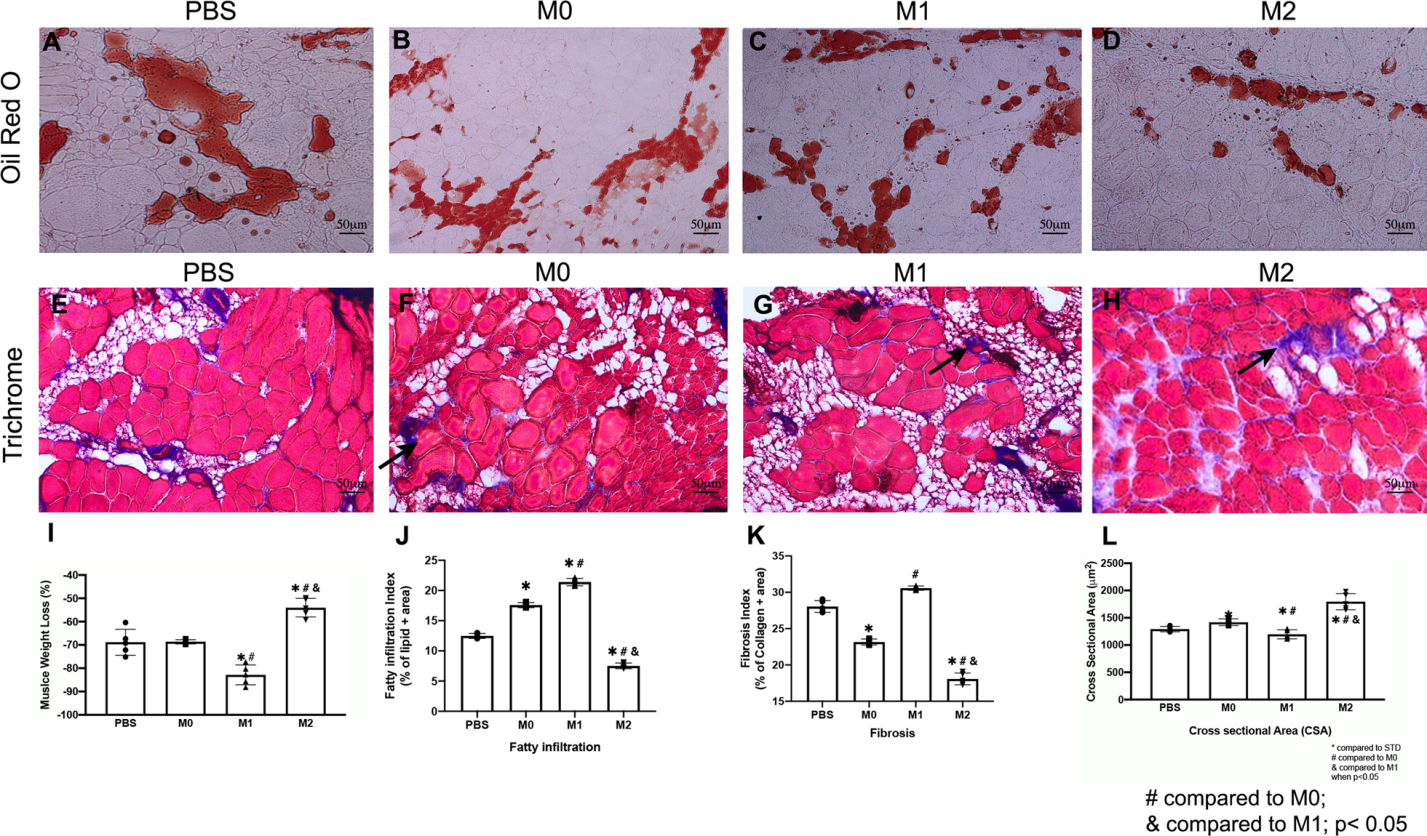
M0 exosome injection diminished muscle regeneration 6 weeks after rotator cuff injury (TT + DN). M2 exosome injection enhanced muscle regeneration after TT + DN surgery. **A**–**D** Typical Oil Red O stained images (original magnification × 200) of SS in PBS, M0, M1, and M2 exosome injection experimental groups (Lipid droplet-Red). **E**–**H** Typical Trichrome staining images of SS in PBS, M0, M1, and M2 exosome injection experimental groups (Collagen—Blue, Muscle Fiber—Red). **I** Muscle weight 6 weeks after rotator cuff injury (TT + DN) normalized with body weight. Muscle weight loss = ([SS right—SS left]/SS left) × 100%. **J** Quantification of the fatty infiltration index was measured as the percentage of the area of lipids out of the total area of the image. **K** The quantification of the fibrosis index was measured as the percentage of the area of collagen out of the total area of the image. **L** The quantification of myofiber cross-sectional area, mean $\pm$ SD (*compared to the PBS group # compared to M0 & compared to M1 when *p* < 0.05)

**Fig. 8:**
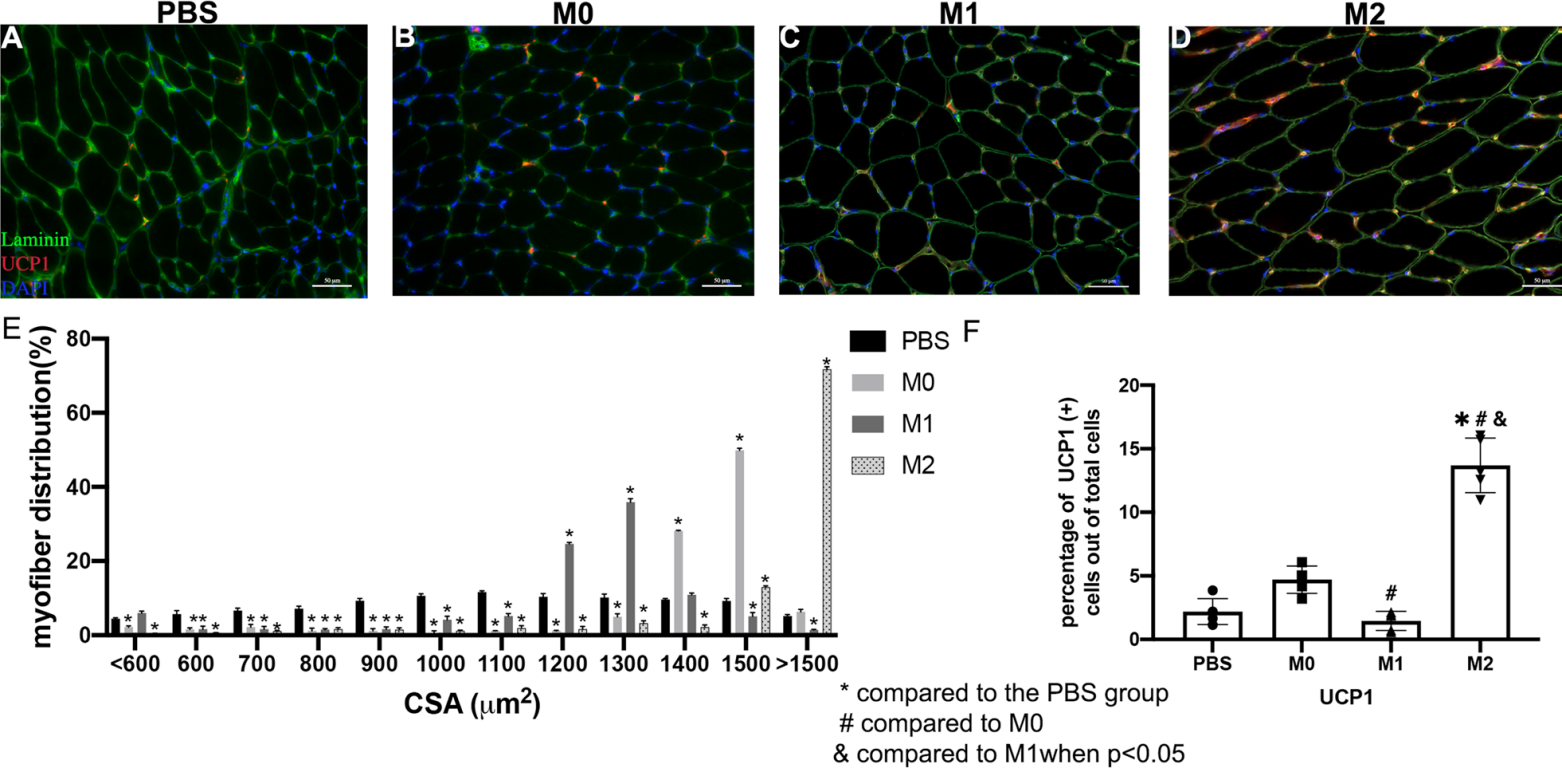
**A**–**D** Typical immunofluorescence images of UCP1 of SS (original magnification × 200) in PBS, M0, M1, and M2 exosome injection groups (UCP1—red, laminin—green, DAPI—blue). **E** The muscle fiber size distribution by percentage (CSA um2). **F** The quantification of UCP1 expression with the percentage of UCP1 positive cells out of the total cells (* *p* < 0.05 compared to the PBS group # compared to M0 & compared to M1 when *p* < 0.05)

Histology analysis showed muscle weight loss, fatty infiltration, and fibrosis significantly decreased in SS muscle after M2 macrophage exosome injection at 6 weeks injury when compared to PBS (Fig. 7). Moreover, the cross-sectional area of SS muscle significantly improved in M2 exosome treatment group when compared to other groups (1794.6 ± 147.2(M2) vs 1196.6 ± 82.7(M1) vs 1418.7 ± 62.3(M0) and 1293.3 ± 50.3 (PBS), *p* = 0.014 Fig. 7). The overall UCP1 expression was significantly increased in the M2 macrophage exosome treatment group (13.7 ± 2.2) when compared to the control (2.4 ± 0.9, *p* = 0.002) (Figs. 7, 8). Injection of M2 macrophage exosomes decreased fatty infiltration, fibrosis, atrophy, and induced UCP1 expression in SS muscle.

## Discussion

In this study, we showed dynamic polarization of infiltrated macrophages in rotator cuff muscles following combined tendon and nerve injury. Infiltration of inflammatory cells has been reported during rotator cuff muscle atrophy and fatty infiltration in previous clinical and pre-clinical studies. In a recent study, Krieger et al*.* analyzed immune cell subsets that infiltrated supraspinatus muscles in a mouse model of rotator cuff injury with tenotomy and/or denervation [17]. At 7 days post-surgery, they found dramatic infiltration of unpolarized and polarized macrophages, dendritic cells, and T lymphocytes in RC muscle after tendon and nerve injury. Combined tendon and nerve injury led to more macrophage infiltration in both M1 and M2 subtypes compared to isolated tendon injury. However, most previous studies of macrophage infiltration in rotator cuff muscles focused on a single time point following injury. In our current study, we observed a shift from a classically activated “pro-inflammatory” M1 macrophage dominant phase to an alternatively activated “pro-regenerative” M2 macrophage dominant phase during the six-week time course following a combined tendon and nerve injury. This data suggests the existence of an early “inflammation” phase and a later “healing” phase of rotator cuff muscle atrophy and fatty infiltration after tendon/nerve injury. These data also suggest that macrophage polarization plays an important role in this process.

FAPs are a group of residential muscle progenitor cells that play a critical role in regulating muscle homeostasis. Previous studies have demonstrated that FAPs are responsible for muscle fibrosis and fatty infiltration following muscle injuries [34, 35]. Our previous study demonstrated that FAPs are the cellular source of fibroblasts and adipocytes seen in rotator cuff muscle fibrosis and fatty infiltration [18]. However, the underlying mechanism of FAP fibrogenesis and adipogenesis during the development of muscle fibrosis and fatty infiltration following rotator cuff injury remains largely unknown. Histological analysis of FAP reporter mice in this study showed physical proximity between macrophages and FAPs in rotator cuff muscles following tendon/nerve injury. An In vitro coculture study further demonstrated that polarized macrophages directly regulate FAP fibrogenesis and adipogenesis. These data suggest that polarized macrophages are important regulators of rotator cuff muscle fibrosis and fatty infiltration.

At the same time, FAPs are also reported to have an important role in muscle regeneration by facilitating satellite cell myogenesis [36, 37]. Previous work, including our own, showed that FAPs can adopt a new brown/beige fat (BAT) differentiation as evidenced by the expression of UCP1, a hallmark of BAT [38]. Though morphologically similar to white fat, beige fat can return to brown-like fat in response to specific stimulation. Because of the inherent plasticity of BAT, inducing “browning” of white adipose tissue has become a new hope for treating type II diabetes, obesity, and other metabolic diseases [39–42]. Indeed, in our previous studies, we have shown that the induction of FAP BAT differentiation with one of the beta-3 adrenergic receptor agonists (Mirabegron) significantly reduced muscle atrophy, fibrosis, and improved shoulder function after RC tears and repair in mice [1, 43]. Beyond their metabolic role, beige/brown adipose tissue (BAT) has been identified as an endocrine organ, which secretes various growth factors, known as batokines, including myogenic trophic factors, that have a role in promoting muscle growth and SC population expansion [44]. In our previous study, we have shown that transplantation of BAT differentiated FAPs improved muscle atrophy, fatty infiltration, and shoulder function after RC tears [45, 46]. In this study, we observed that M2 macrophages and their derived exosomes induced FAP BAT differentiation in vitro and in vivo. We further showed that transplantation of M2 macrophages significantly reduced muscle atrophy, fibrosis, and fatty infiltration in RC muscles, as well as increased FAP BAT differentiation. These data suggest that inducing FAP BAT differentiation may be an important underlying mechanism of M2 macrophage mediated mitigation of RC muscle atrophy and fatty infiltration.

Exosomes are membrane vesicles with a diameter of 30–100 nm that are secreted by cells into the extracellular milieu. Because exosomes contain messenger molecules as cargo that play an important role in regulating intercellular signaling, cell behavior, and cell function, the therapeutic application of these “bioactive vesicles” for treating various diseases has gained increased attention. For example, exosomes have been used as new therapeutics for cancer [47], autoimmune diseases [48], ischemic stroke [49], Alzheimer’s disease [50], osteoarthritis [51] and other diseases. Compared to cell-based therapies, exosome therapy has similar substantial protective effects with a decrease in potential tumorigenic and immunogenic side effects [52]. In a recent study, Wang et al. used exosomes from adipose-derived stem cells (ASC) to treat rotator cuff muscle atrophy and fatty infiltration in rats. They found ASC-derived exosomes significantly reduced RC muscle atrophy, fatty infiltration and inflammation following massive tendon tears [53]. This suggests the feasibility in treating RC muscle atrophy and degeneration with exosomes. In our study, we found that M2 macrophage-derived exosomes have a similar effect in reducing RC muscle atrophy, fibrosis, and fatty infiltration in mice. M2 macrophage-derived exosomes may serve as a new option for treating rotator cuff muscle atrophy and fatty degeneration in the future.

There are several limitations in our study. First, many inflammatory cells other than macrophages (such as dendritic cells and T lymphocytes) were reported in RC muscle after tendon tears [17]. However, we only evaluated the role of macrophages in this study. A previous study showed that T lymphocytes are not required for the development of fatty degeneration after rotator cuff tear [54]. Thus, T-lymphocytes may not play an important role in regulating FAP differentiation. Future work is warranted to investigate the role of dendritic cells in FAP regulation and RC muscle fatty infiltration. Second, although we proved the functional role of polarized macrophage-derived exosomes in regulating FAP differentiation and RC muscle atrophy and fatty infiltration, the effective components in macrophage-derived exosomes are not defined in this study. In a previous study, we reported that micro-RNA-99a/146b/378a was the active cargo in M2 macrophage-derived exosome responsible for resolving atherosclerosis [25]. Future work is needed to determine if those miRNAs are equally important in regulating FAP differentiation and RC muscle degeneration. Third, only young female C57BL/6 J mice were used for macrophage and their exosome transplantation experiments due to time and budget constraints. Future large-scale work will allow us to address sex, age, and strain differences in response to exosome treatment. Finally, data from mice in this study need to be validated in human cells and muscles before being translated into human studies.

## Conclusions

In conclusion, our results from this study showed that polarized macrophages directly regulate FAP differentiation through their exosomes. M2 macrophage-derived exosomes may serve as a novel treatment option for muscle atrophy and fatty infiltration.

### Supplementary Information

**Additional file 1: Fig. S1.** Flow cytometry gate for FAP isolation. FAPs were characterized with CD31-/CD45-/ITG7-/Sca1+/CD140a+ population within the muscle.

**Additional file 2: Fig. S2.** **A** Exosomes (larger spots) were derived from M0-polarized macrophages. The exosomes have a visible lipid bilayer (donut shape). The smaller spots are Uranyl Acetate stain artifacts. **B** Exosomes (larger spots) were derived from M1-polarized macrophages. **C** Exosomes (larger spots) were derived from M2-polarized macrophages. **D** The nanosight of exosomes isolated from M0, M1 and M2 cultured media. **E** Particles per million cells in the M0, M1 and M2 cultured media. **F** The average mode of size of exosomes isolated from M0, M1 and M2 cultured media. **G** The average concentration of particles isolated from M0, M1 and M2 cultured media.

**Additional file 3: **Schematic flow of the experimental design.

**Additional file 4: Table S1.** The primer sequences used in this experiment was listed.

## Data Availability

The datasets used and or analyzed during the current study are available from the corresponding author on reasonable request.

## Abbreviations

FI: Fatty infiltration
KO: Knockout
FAPs: Fibro/adipogenic progenitors
TGFβ: Transforming growth factor beta
WT: Wild type
BMP: Bone morphogenetic protein
αSMA: Alpha smooth muscle actin
RC: Rotator cuff
SS: Supraspinatus
EV: Extracellular vesicles
MVB: Multivesicular endosomal compartment
lncRNAs: Long non-coding RNAs
IFNγ: Interferon gamma
C-DGUC: Cushioned-density gradient ultracentrifugation
FBS: Fetal bovine serum
BAT: Brown/beige fat
UCP1: Uncoupled protein 1
PRDM16: PR/SET domain 16
Dio2: Type II iodothyronine deiodinase
PPARγ-1: Peroxisome proliferator-activated receptors gamma-1
PDGFRα-GFP: Platelet-derived growth factor receptor alpha-green fluorescence protein
PBS: Phosphate Buffer solution
TT + DN: Tendon transection plus suprascapular denervation transection
ASC: Adipose-derived stem cells
