# Supplementary figures and images for: Polarized macrophages regulate fibro/adipogenic progenitor (FAP) adipogenesis through exosomes
Source: Stem Cell Res Ther. 2023 Nov 7;14:321. doi: 10.1186/s13287-023-03555-6 (PMC10631219; doi:10.1186/s13287-023-03555-6)

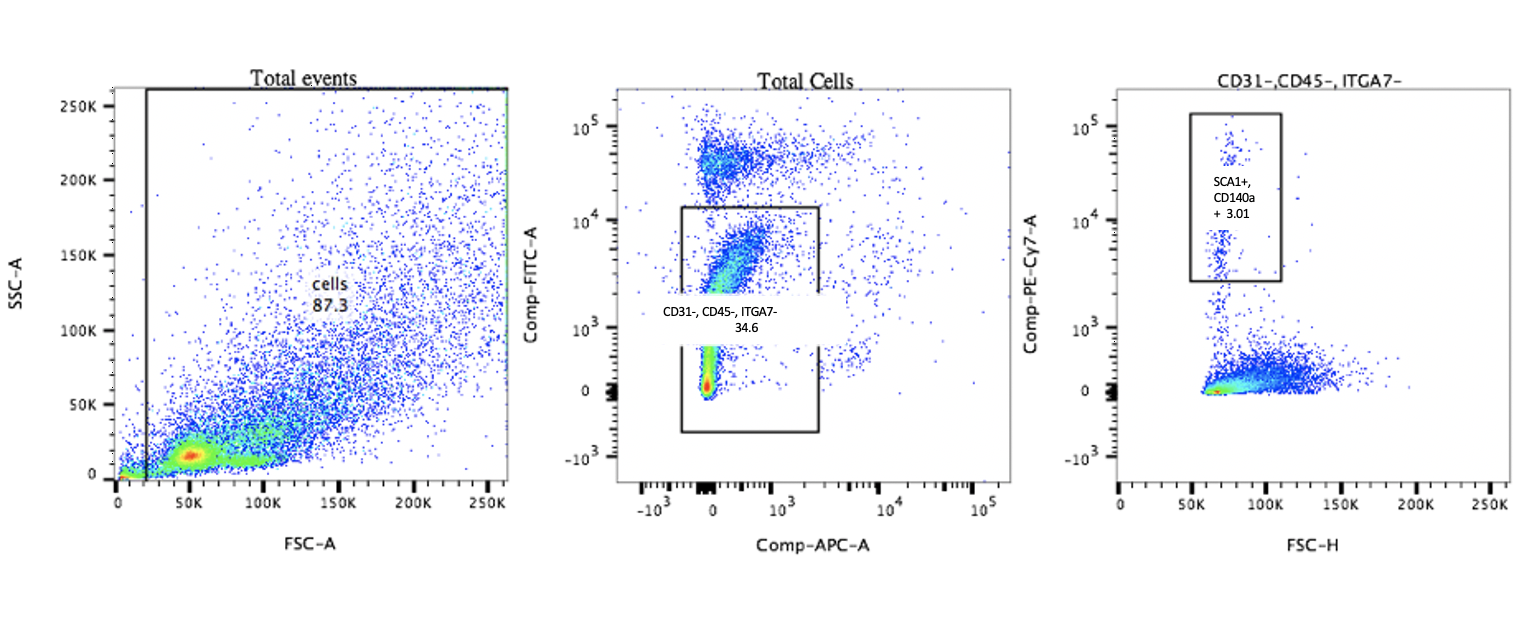

Supplement: Supplementary file 1 — Additional file 1: Fig. S1. Flow cytometry gate for FAP isolation. FAPs were characterized with CD31-/CD45-/ITG7-/Sca1+/CD140a+ population within the muscle. [file 13287_2023_3555_MOESM1_ESM.tiff]

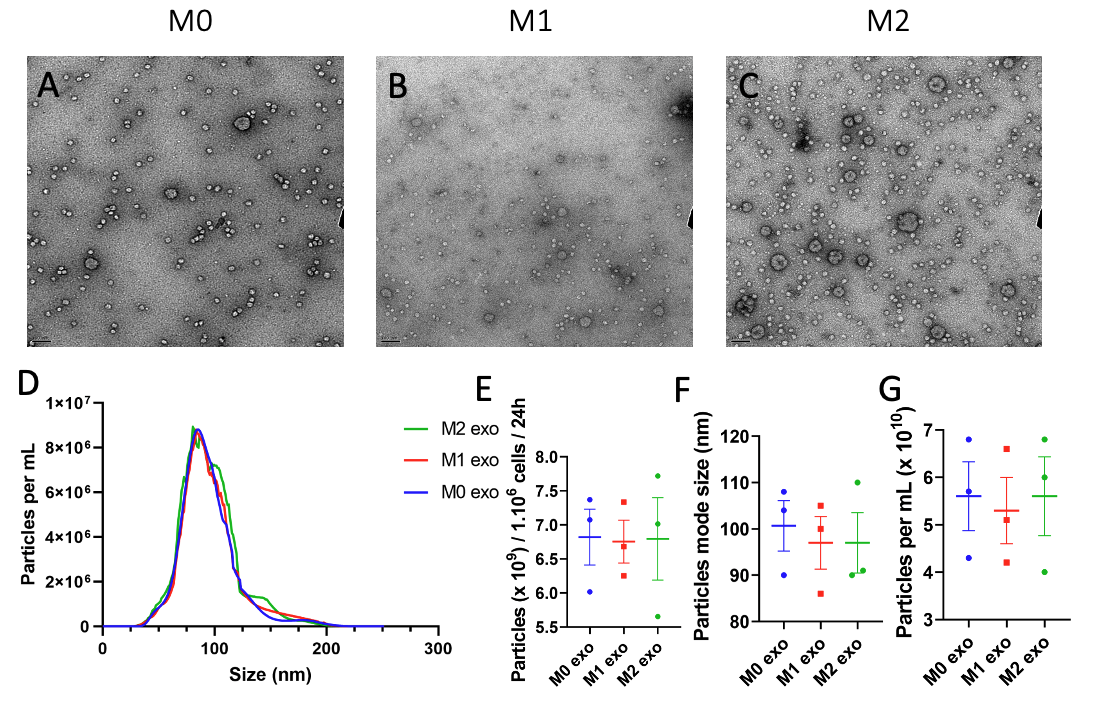

Supplement: Supplementary file 2 — Additional file 2: Fig. S2. A Exosomes (larger spots) were derived from M0-polarized macrophages. The exosomes have a visible lipid bilayer (donut shape). The smaller spots are Uranyl Acetate stain artifacts. B Exosomes (larger spots) were derived from M1-polarized macrophages. C Exosomes (larger spots) were derived from M2-polarized macrophages. D The nanosight of exosomes isolated from M0, M1 and M2 cultured media. E Particles per million cells in the M0, M1 and M2 cultured media. F The average mode of size of exosomes isolated from M0, M1 and M2 cultured media. G The average concentration of particles isolated from M0, M1 and M2 cultured media. [file 13287_2023_3555_MOESM2_ESM.tiff]

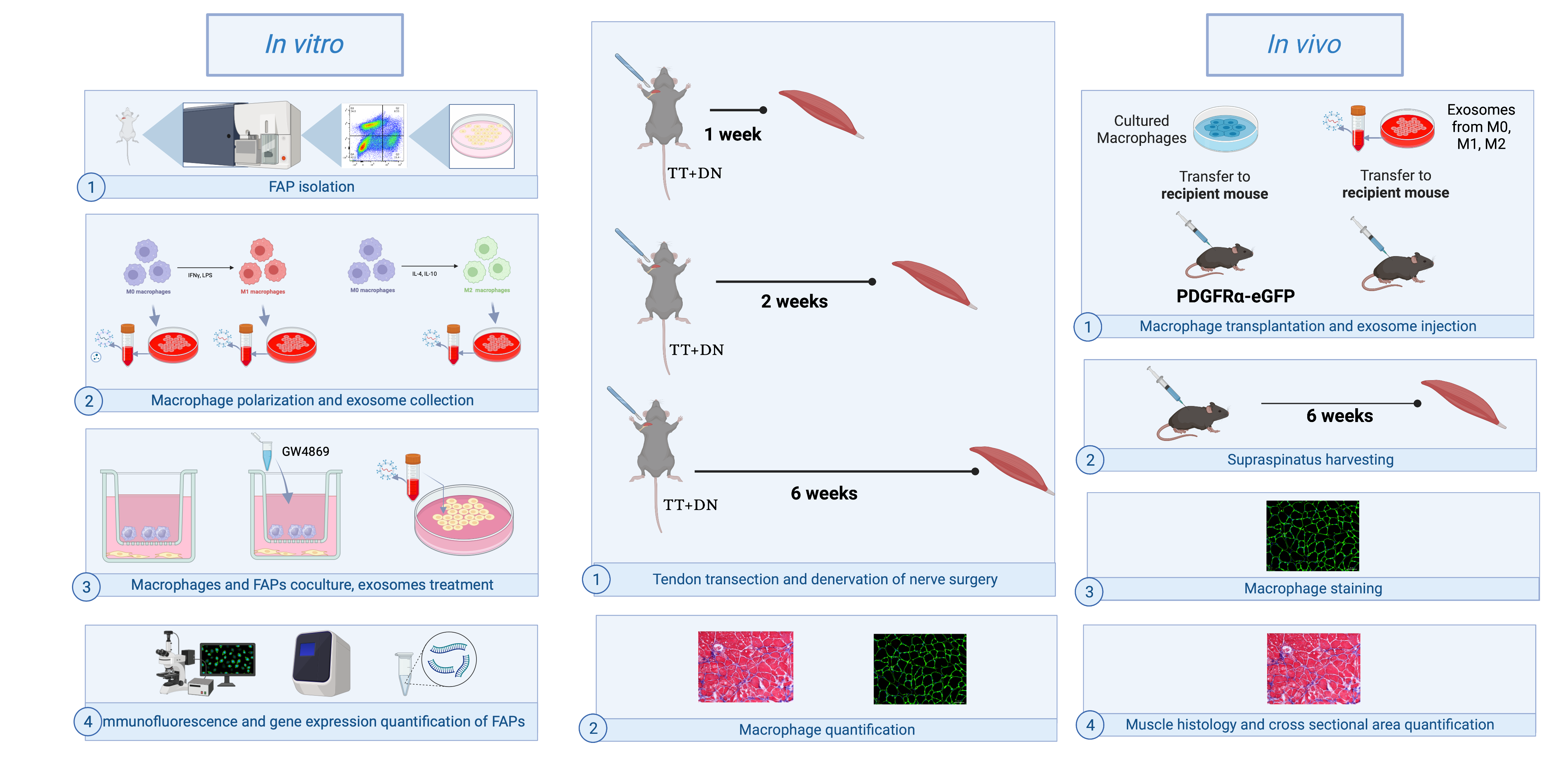

Supplement: Supplementary file 3 — Additional file 3: Schematic flow of the experimental design. [file 13287_2023_3555_MOESM3_ESM.png]

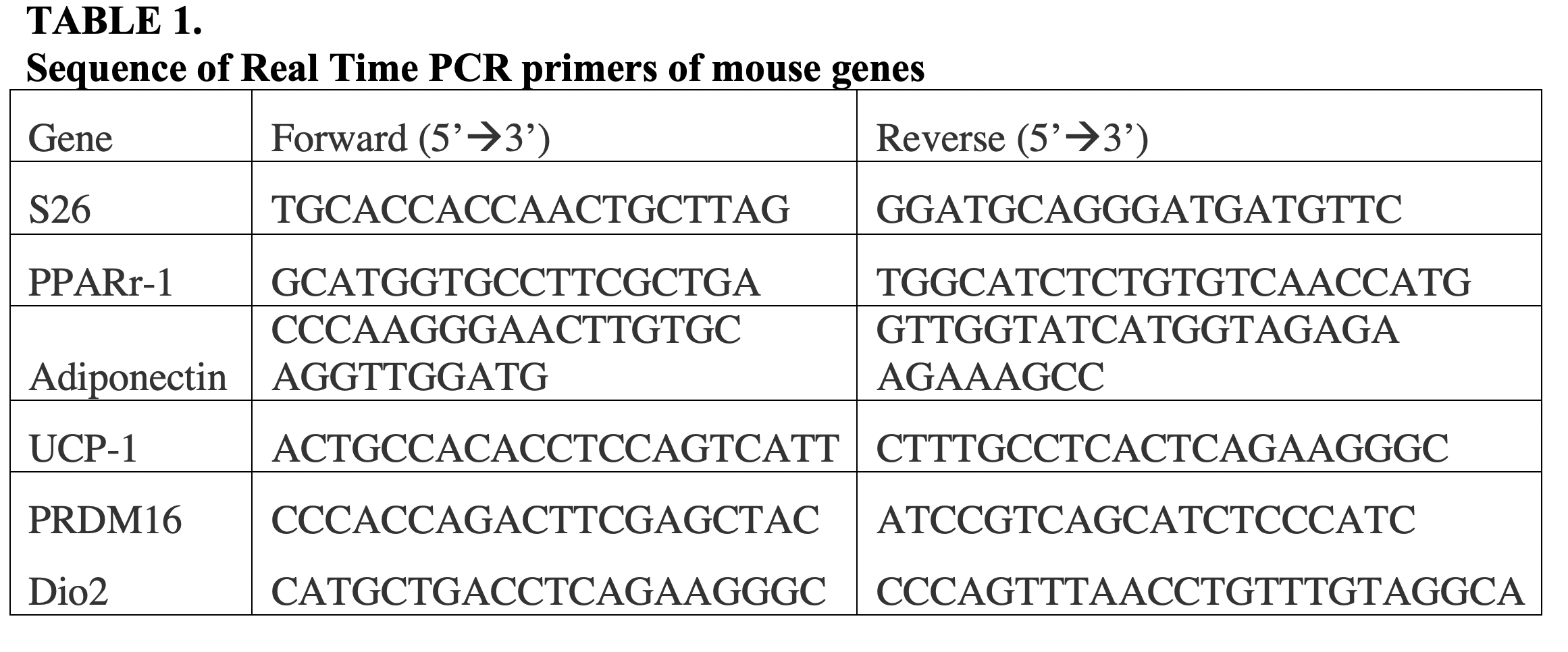

Supplement: Supplementary file 4 — Additional file 4: Table S1. The primer sequences used in this experiment was listed. [file 13287_2023_3555_MOESM4_ESM.png]
